# Illinois State Medical Society

**Published:** 1881-07

**Authors:** 


### Society Reports.

### Article VI.

Illinois State Medical Society. Thirty-first Annual Meeting held May 17, 18 and 19, 1881, in the Methodist Church Block, Chicago. Dr. George Wheeler Jones, of Danville, President, in the chair; Dr. S. J. Jones, of Chicago, Permanent Secretary.

### First Day—May 17.

This was one of the largest meetings ever held by the Society, whose membership numbered 114, but the audience was reinforced by an unusually large number of visitors from the city, and this and other states. The

PRESIDENT'S ADDRESS

was decidedly conservative in character, and strong in its denunciation of the many abuses which had crept into the profession at large. Effectual medical legislation seemed an almost impossible achievement, and he denounced the present medical training as inadequate to the exigences of modern practitioners of medicine, and advised a graded system of education.

The report of the Committee on Practice of Medicine was read by the Chairman, Dr. E. P. Cook, of Mendota. It covered the time from May, 1880, to the date of this meeting, and consisted of reports from the various parts of the State. It was thought that no important phase of any epidemic had been overlooked, but there was much difficulty in getting the particulars of each, and a more efficient form of correspondence ought to be established between the various members. The appointment of correspondents from the local societies through Illinois might answer this purpose. Nothing unusual had happened until October,

1880, except a slight increase in malarial diseases, but from that time these and many other zymotic diseases had prevailed to quite an extent. The protracted winter from which we had just emerged was doubtless a powerful factor in their causation. The southern part of the State had been more favored. The measles prevailed in every county. Scarlatina and diphtheria prevailed in an epidemic form in some of the central and northern counties. Cerebro-spinal fever complicated some cases of measles. Small-pox had extended from Chicago to most parts of the State. Pneumonia had also been unusually frequent, and an epidemic of dysentery had been observed. The mortality from whooping-cough, and that from erysipelas had been greater than usual. Winter cholera had prevailed in Chicago ; it was so mild as to escape the observation of many. A few cases of trichinosis had been reported, but probably many more had been overlooked, as was generally the case.

Report of Dr. Charles T. Parkes, of Chicago, Chairman of the *Committee on Surgery.*

### TREATMENT OF WOUNDS.

Free drainage, close coaptation, and perfect rest are requisites. The first was obtained by the use of rubber tubes, or what was better, absorbable tubes made of chicken bones. Silk-worm guts form an advantageous substitute to horse hair in capillary drainage. To insure coaptation, the cat-gut ligature was very good, but as it was soon absorbed it might give rise to secondary hæmorrhage, and torsion was still preferred in some hospitals in the arrest of hæmorrhage. Cat-gut soaked fourteen days in one part of chromic acid to thirty of glycerine was so hardened as not to be so readily absorbed. Silk-worm guts were a good substitute. Rest of a wounded limb should be secured by the use of splints in some cases.

Listerism was now adopted by most of the younger surgeons, and when the spray was dispensed with, the washes with carbolic acid solutions were used. Lister's dressing also contributed to insure perfect rest of the wounded parts. The presence of bacteria in suppurating wounds had been often detected, and when injected into healthy tissues gave rise to abscesses, but under the use of the spray, these bacteria had disappeared from wounds.

The dressings should be renewed often enough to prevent any irritation to the parts from retained fluids.

VARICOCELE.

The treatment of this disease was very unsatisfactory, frequent cases of death or of permanent injury occurred, and Dr. R. G. Bogue had made quite a departure from the common treatment, in opening the scrotum antiseptically, ligating a part of the veins at a time with cat-gut, and closing the wounds. This had given most satisfactory results, and could be applied also to varicose veins in any part of the body.

NERVE RESECTION AND STRETCHING.

The Chairman had made an efficient resection of the ulnar nerve in two instances, and sensation and motion were re-established after the extremities of the nerve had been pared and brought together with cat-gut ligatures. Nerve stretching was considered a favorable operation in neuralgias, tetanus, epilepsy, locomotor ataxia, and other diseases. Experiments to ascertain the tension of nerve trunks in men were made on recent subjects, with the following results: Eighty pounds break the ulnar nerve; 57, the median; 18, the radial; 61, the brachial; 7½, the facial; 200, the great sciatic; 92, the cauda equina. Dr. Parkes thought he had been the first to institute such experiments.

A case of non-union after a compound fracture of the tibia in an old man, had come under the observation of Dr. D. S. Booth, of Sparta. The foot was useless and amputation seemed proper.

Dr. W. A. Byrd supplemented the report on Surgery with some very interesting cases. In one he had trephined into the frontal sinuses of a patient, and removed some decayed bone which had caused a supra-orbital neuralgia of fifteen years duration. He had made tracheotomy without a tube, replacing it by wires. He related a new method of nerve section in facial neuralgia, and exhibited some surgical instruments remarkable for their simplicity, cheapness and usefulness.

*Committee on Obstetrics.*—Dr. H. Webster Jones, Chairman, read the report of that committee, and said that the accoucheur should inspire confidence, he should palliate suffering, supplement nature, and attend to any complication. His hand was the best uterine dilator. Forceps should not be used unless strongly indicated. Long delay in labor was a common cause of inflammation and blood poisoning. The delivery of the placenta should be facilitated by placing one hand over the uterus externally. A careful examination should be made six weeks after parturition to satisfy the accoucheur as to the integrity of the various organs of generation.

Dr. De Laskie Miller laid great stress on the necessity for the accoucheur to be learned and skillful. He gave some rules for dilating the os with the fingers and tearing any adhesion of the membranes to the uterus. He said that rupture of the perineum could be avoided if the attendant had a proper knowledge of the anatomy of the parts, and made sufficient support.

Dr. J. W. Dora read the report of the *Committee on Gynæcology.*

He complained that this is the only specialty which was not practically taught to students. He hoped for the time when false modesty would no longer interfere with clinical demonstrations. He denounced several modes of treatment which had been fashionable in their days and caused many bad results. His treatment of obstructive dysmenorrhœa consisted in making an incision and introducing sponge tents dipped in a carbolic acid solution. It needed to be used repeatedly, but it would generally prove successful. He gave rules for the treatment of rupture of the perineum.

Dr. Ellen A. Ingersoll read a supplementary paper on *Dysmenorrhœa.*

It was often caused by displacement, but the majority of cases could be referred to a congestive condition of the uterus. It was liable to occur soon after the age of puberty, and to disappear after repeated pregnancies. The general health was often below par. The function of the liver in excreting cholesterine, the detritus of nerve substance, was ill-performed in those cases, and constipation was a frequent accompaniment. Mercurials were especially suited to these. Alteratives, tonics, and hygienic measures were beneficial. For an immediate relief German chamomile, aromatic spirits of ammonia and viburnum were as good as any other remedy. The introduction of a sponge tent a few days before the return of the menses prevented dysmenorrhœa.

SECOND DAY—MAY 18.

Dr. F. C. Hotz, of Chicago, read the report on *Ophthalmology and Otology.*

Overdoses of quinine had produced blindness as well as deafness. In a case of keratitis, quinine had brought relief after local treatment had proven useless. Homatropine was an efficient dilator of the pupil, and its effects soon passed away. The report was unfavorable to the audiphone. Dr. Hotz gave the particulars of a case of malarial otitis which subsided after the use of quinine in scruple doses. Enucleation of the eye-ball could not be replaced by section of the nerves, as these would unite soon and sympathetic inflammation would continue.

*Committee on Dermatology.*—Dr. W. J. Maynard, of Chicago, read the report. His subject was ring-worm of the scalp. Its proportion to other diseases was one to thirty. Its diagnosis required time and skill, and so its treatment. It was quite liable to cause baldness. Ring-worm of the body was a common affection, but it passed unobserved. The type of the disease was lost after some time, and would readily be mistaken for eczema. The cause of the disease was a microscopic organism, which gave rise to the same in some of the domestic animals. The best treatment consisted in mercurials, sulphurous acid, iodine and carbolic acid. Epilation was necessary in many cases, and this was done with large forceps. Iron, arsenic, iodide of potassium were given internally with cod-liver oil.

Dr. C. T. Reber, of Shelbyville, reported two cases of submucous fibroids complicating pregnancy. There had been abundant hæmorrhage in both cases, but they had reached full term.

Dr. W. S. Caldwell, of Freeport, read a paper on the management of the after-birth in abortion. No great amount of hæmorrhage should be allowed, but the hand or the fingers should be introduced and the placenta removed. He did not commend the use of placental forceps, nor the administration of ergot. He had used the tampon and laminaria tents successfully in some cases.

Dr. E. Fletcher Ingals, of Chicago, read a valuable report on *Laryngeal Tumors*, which appears in full in this number of THE CHICAGO MEDICAL JOURNAL AND EXAMINER.

Drs. Truesdell and Whitmire recommended the use of the carbolic acid inhaler in chronic laryngitis and other affections of the air passages.

ANIMAL HEAT.

Dr. J. H. Hollister, Chicago, read an able paper on that subject. Heat was produced by the vital activity of the animal as much as by chemical changes taking place in the tissues. There was more heat generated in health, but its retention in disease gave rise to fever. He believed there were vaso-dilator nerves, whose primary center was situated in the medulla oblongata, which some medicines influenced, thereby diminishing heat. He also enumerated the various results of a high temperature. Withdrawing food, applying cold to the surface of the body, and administering some cardiac sedatives, reduced the temperature in fever. The cold pack, sponge bath, and ice internally, were especially recommended.

Dr. Hill, of Bloomington, exhibited an instrument invented by Dr. Russell, of Bushnell. It resembled a trocar and canula and was to be introduced into the trachea to replace tracheotomy.

Dr. Maxwell read a paper on *intra-capsular fracture*, and reported what seemed to be two cases of that injury in his own practice. He had made extension in the direction of the limb, and he had made extension transversely. He had also elevated the bed at the foot and on one side. The treatment had also been successful in both cases, and union was perfect.

SPECIALISM IN MEDICINE.

Dr. A. Reeves Jackson, of Chicago, said, in an able paper with the above title, that specialism was nothing new. That there were physicians in ancient Egypt who made a specialty of the treatment of the liver, the skin, the head, and the toe-nails. Multiplicity of professorships and a lack of medical education were the chief causes of specialisms. The consciousness of knowing very little of general practical medicine leads the graduate to work a specialty for himself. He remonstrated against the theoretical teachings of the various colleges, and said that medicine was chiefly an art which could be learned through practice only.

The advantage of specialism was that by concentrating it increased one's energy in a certain direction; that all great men had been specialists, and that it was for such a division of labor that medicine had progressed so rapidly of late. He called professional quacks those that studied a special branch before they had been general practitioners. These would be especially liable to refer all diseases to one organ in particular. His conclusion was that specialism reduced the number of general practitioners without diminishing the practice of the latter, and specialists relied on the profession for its support, but they must have been good common physicians before entering the field of specialism.

Dr. Earle read an article on rötheln, an abstract of which appeared in the last number of THE JOURNAL AND EXAMINER.

Dr. Roswell Park reported 100 cases of the same disease which had come under his observation at the Orphan Asylum.

### THE LEGAL REGULATION OF THE PRACTICE OF MEDICINE.

Dr. M. A. McClelland, of Knoxville, had prepared a report on the above subject, which was read by the Secretary. It was a statement of the various attempts at making efficient legal regulations, with as many failures.

### INFECTIOUSNESS OF TUBERCULOSIS.

Dr. H. Gradle summed up the late experiments made to ascertain the contagiousness of tuberculosis in rabbits. There was no doubt as to the relationship between scrofula, tuberculosis, and fungous arthritis. The incubation of the disease in rabbits was twenty days. Tuberculosis is probably often transmitted through the breath of the patient. A solution of tuberculous matter in a spray inoculated the disease in dogs in the space of three weeks. In all probability the virus was a micro-organism.

Dr. E. F. Ingals said that it was a matter of surprise to him that the relations of bacteria to tuberculosis had not yet been determined. He had no doubt as to the contagiousness of tuberculosis, but he thought another decade would pass before the character of this contagion could be ascertained.

### USES AND ABUSES OF ALCOHOL.

Dr. E. Ingals read a report made up of the opinions of most of the prominent members of the Society. Most of them agreed that alcohol should never be used except as a remedy. That in some cases it was one of the best articles in the Pharmacopœia, and a good stimulant. It was a narcotic poison and an anæsthetic in large doses. Drunkenness was a vice of advanced civilization, which should be combatted by the example and the principles of the medical profession.

### THIRD DAY—MAY 19.

The first paper in order was one on Chian Turpentine, by Dr. E. Andrews, of Chicago. This remedy had proved useless in the treatment of any variety of cancer. A great many cases of cancer of the womb, of the breasts, etc., all of them following the usual course of the disease, were related, and the conclusion was reached that Chian turpentine did not deserve a better place than any other remedy in the therapeutics of cancer.

#### EPILATION.

Dr. Plym. S. Hayes, of Chicago, read an interesting paper on this subject. He claimed the best depilatory was electrolysis; which he performed thus: A needle was attached to the negative pole and plunged into the hair follicle, the hair being held by forceps; the other pole was placed near the spot, and the current destroyed the hair follicle. It required little traction to pull the hair, which could never grow again. This process was not painful, the electrolysis caused some anæsthesia of the parts. It seldom needed to be repeated.

#### ANATOMY OF THE SHEATHS OF THE PALMAR TENDONS.

Dr. Roswell Park read a contribution with this title, an abstract of which was printed in the last number of THE JOURNAL AND EXAMINER.

#### LISTERISM AND CARBOLIC ACID.

Dr. C. Truesdell read a report largely composed of the views of many surgeons on this subject. He reported a case of mummification of the finger from pure carbolic acid. It had been painless. He thought this remedy serviceable, especially on account of its antiphlogistic properties, and considered it the best local application to wounds and ulcers. Dr. J. P. Johnson, of Peoria, had used phenol with as good results as carbolic acid. He was especially gratified with the use of carbolic acid as a cauterizing agent. It possessed the great advantage over all other acids that it is painless in its application. He had cured ulcers in this manner, and had successfully treated endometritis by the application of carbolic acid to the inside of the uterus. He was in the habit of applying it with a stick fashioned as desired.

The Committee on State Register had been satisfied that the Register of the State Board of Health entirely fulfilled the wants of the Society. The committee was discharged.

The Treasurer's report showed a very prosperous condition of affairs.

The *Committee on Necrology* reported the death of Dr. F. H. Davis, son of N. S. Davis, of Chicago.

A resolution was passed providing that members who had paid their annual dues during fifteen years should not be suspended for non-payment of the same in the future, but that they would not be entitled to the Annual Transactions.

A paper on "Drainage of Cavities in the Lungs," by Drs. C. Fenger and J. H. Hollister, of Chicago, was presented to the Society, and referred to the Committee on Publication, with all the other papers read before this meeting.

ELECTION OF OFFICERS FOR THE PRESENT YEAR.

*President*—Dr. Robert Boal, Peoria.

*First Vice President*—Dr. A. T. Darrah, Tolono.

*Second Vice President*—Dr. Ellen A. Ingersoll, Canton.

*Treasurer*—Dr. J. H. Hollister, Chicago.

*Secretary*—Dr. W. A. Byrd, Quincy.

*Committee of Arrangements*—Drs. Jas. Robbins, J. T. Wilson, F. Drude, J. A. Wagner, M. F. Bassett.

STANDING COMMITTEES.

*Practical Medicine*—Dr. W. D. Ensign, Rutland, Chairman; Dr. John Wright, Clinton; Dr. H. T. Godfrey, Galena.

*Surgery*—Dr. E. W. Lee, Chicago, Chairman; Dr. D. W. Graham, Chicago; Dr. J. P. Mathews, Carlinville.

*Obstetrics*—Dr. J. B. Davison, Malone, Chairman; Dr. A. C. Corr, Carlinville; Dr. J. Y. Campbell, Paxton.

*Gynæcology*—Dr. E. W. Jenks, Chicago, Chairman; Dr. G. W. Nesbitt, Sycamore; Dr. F. Cole, El Paso.

*Ophthalmology and Otology*—Dr. A. E. Prince, Jacksonville, Chairman; Dr. W. T. Montgomery, Chicago; Dr. C. Chenowith, Decatur.

*Drugs and Medicines*—Dr. Joseph Robbins, Quincy, Chairman; Dr. T. J. Pitner, Jacksonville; Dr. L. L. Silverthorne, Charleston.

*Necrology*—Dr. E. Ingals, Chicago, Chairman; Dr. W. Hill, Bloomington; Dr. W. West, Belleville.

SPECIAL COMMITTEES.

*Relations Between Syphilitic and Non-Syphilitic Lesions of the Skin*—Dr. James Nevins Hyde, Chicago.

*On the Management of Diseases of Children*—Dr. W. O. Mendenhall, Georgetown.

*On Medical Legislation*—Dr. W. A. McClellan, Knoxville, Chairman; Dr. D. S. Booth, Sparta; Dr. W. Hill, Bloomington; Dr. C. Truesdell, Rock Island; Dr. E. Ingals, Chicago.

*On Diseases of the Nervous System*—Dr. S. M. Wylie, Paxton.

MEMBERS OF JUDICIAL COUNCIL.

Dr. C. Truesdell, Rock Island; Dr. C. Goodbrake, Clinton; Dr. A. K. Van Horne, Jerseyville.

The next annual meeting will take place at Quincy.

*Delegates to the American Medical Association*, to meet at St. Paul in June, 1882.—Drs. J. H. Hollister, Chicago; W. T. Kirk, Atlanta; W. T. Montgomery, Chicago; G. W. Jones, Danville; E. P. Cook, Mendota; A. Hard, Aurora; R. Boal, Peoria; G. W. Nesbitt, Sycamore; Sarah H. Stevenson, Chicago; J. M. Cowan, Hennepin, W. R. Shinn, Fielding; W. M. Call, Princeton; Catharine B. Slater, Aurora; J. P. Johnson, Peoria; John R. Livingood, Rossville; J. S. Whitmire, Metamora; S. T. Hurst, Greenview; Ellen A. Ingersoll, Canton; D. Lichty, Rochelle; D. E. Foote, Belvidere; D. S. Jenks, Plano; R. G. Bogue, Chicago; T. D. Washburn, Hillsboro; C. Truesdell, Rock Island; A. H. Kinnear, Metamora; David Prince, Jacksonville; E. W. Jenks, Chicago; W. West, Belleville; H. P. Newman, Chicago; D. S. Booth, Sparta; W. H. Fitch, Rockford; Thomas Galt, Rock Island; W. L. Ransom, Roscoe; J. W. Dora, Mattoon.

*To the Wisconsin State Medical Society*—Dr. Roswell Park, Chicago.

*To the Indiana State Medical Society*—W. O. Mendenhall, Georgetown; W. M. Chambers, Charleston.

*To the Ohio State Medical Society*—J. R. Corbus, La Salle; Daniel Lichty, Rochelle.

*To the Michigan State Medical Society*—J. H. Hollister, Chicago; E. W. Jenks, Chicago.

*To the Kentucky State Medical Society*—M. W. Hurst, Sweet Water.

*To the Missouri State Medical Society*—H. W. Morehouse, Danville.

*To the Iowa State Medical Society*—M. A. McClellan, Knoxville; S. L. Plummer, Rock Island.

*To the Minnesota State Medical Society*—B. F. Crummer, Warren.

*To the International Medical Congress*, to meet in London in August, next—Drs. R. N. Isham, Chicago; De Laskie Miller, Chicago; S. J. Jones, Chicago; W. A. Haskell, Alton; David Prince, Jacksonville.

---

